# The combination of dextran sulphate and polyvinyl alcohol prevents excess aggregation and promotes proliferation of pluripotent stem cells in suspension culture

**DOI:** 10.1111/cpr.13112

**Published:** 2021-08-13

**Authors:** Xianglian Tang, Haibin Wu, Jinghe Xie, Ning Wang, Qicong Chen, Zhiyong Zhong, Yaqi Qiu, Jue Wang, Xiajing Li, Ping Situ, Liangxue Lai, Mark A Zern, Honglin Chen, Yuyou Duan

**Affiliations:** ^1^ School of Biomedical Sciences and Engineering Guangzhou International Campus South China University of Technology Guangzhou China; ^2^ Laboratory of Stem Cells and Translational Medicine Institutes for Life Sciences and School of Medicine South China University of Technology Guangzhou China; ^3^ Guangzhou First People's Hospital School of Medicine South China University of Technology Guangzhou China; ^4^ Key Laboratory of Regenerative Biology South China Institute for Stem Cell, Biology and Regenerative Medicine Guangzhou Institutes of Biomedicine and Health Chinese Academy of Sciences Guangzhou China; ^5^ Department of Internal Medicine University of California Davis Medical Center Sacramento CA USA; ^6^ National Engineering Research Center for Tissue Restoration and Reconstruction South China University of Technology Guangzhou China; ^7^ Key Laboratory of Biomedical Engineering of Guangdong Province South China University of Technology Guangzhou China; ^8^ Key Laboratory of Biomedical Materials and Engineering of the Ministry of Education South China University of Technology Guangzhou China; ^9^ Innovation Center for Tissue Restoration and Reconstruction South China University of Technology Guangzhou China

**Keywords:** cell aggregates, dextran sulphate, human pluripotent stem cells, polyvinyl alcohol, spinner flask, suspension culture

## Abstract

**Objectives:**

For clinical applications of cell‐based therapies, a large quantity of human pluripotent stem cells (hPSCs) produced in standardized and scalable culture processes is required. Currently, microcarrier‐free suspension culture shows potential for large‐scale expansion of hPSCs; however, hPSCs tend to aggregate during culturing leading to a negative effect on cell yield. To overcome this problem, we developed a novel protocol to effectively control the sizes of cell aggregates and enhance the cell proliferation during the expansion of hPSCs in suspension.

**Materials and Methods:**

hPSCs were expanded in suspension culture supplemented with polyvinyl alcohol (PVA) and dextran sulphate (DS), and 3D suspension culture of hPSCs formed cell aggregates under static or dynamic conditions. The sizes of cell aggregates and the cell proliferation as well as the pluripotency of hPSCs after expansion were assessed using cell counting, size analysis, real‐time quantitative polymerase chain reaction, flow cytometry analysis, immunofluorescence staining, embryoid body formation, teratoma formation and transcriptome sequencing.

**Results:**

Our results demonstrated that the addition of DS alone effectively prevented hPSC aggregation, while the addition of PVA significantly enhanced hPSC proliferation. The combination of PVA and DS not only promoted cell proliferation of hPSCs but also produced uniform and size‐controlled cell aggregates. Moreover, hPSCs treated with PVA, or DS or a combination, maintained the pluripotency and were capable of differentiating into all three germ layers. mRNA‐seq analysis demonstrated that the combination of PVA and DS significantly promoted hPSC proliferation and prevented cell aggregation through improving energy metabolism‐related processes, regulating cell growth, cell proliferation and cell division, as well as reducing the adhesion among hPSC aggregates by affecting expression of genes related to cell adhesion.

**Conclusions:**

Our results represent a significant step towards developing a simple and robust approach for the expansion of hPSCs in large scale.

## Graphical abstract

Polyvinyl alcohol (PVA) significantly promotes hPSC proliferation without compromising their pluripotency and differentiation capacity. The combination of PVA and dextran sulphate (DS) enhances cell proliferation and prevents cell aggregation of hPSCs in suspension culture. DS prevents cell aggregation of hPSCs by affecting expression of genes related to cell adhesion. PVA promotes hPSC proliferation by improving energy metabolism‐related processes.

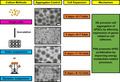

## 1 INTRODUCTION

Human pluripotent stem cells (hPSCs), including human embryonic stem cells (hESCs) and human‐induced pluripotent stem cells (hiPSCs),^1^ exhibit great potential in regenerative medicine and pharmaceutical studies. hESCs, derived from the cell mass within the blastocyst before implantation,^2^ and hiPSCs generated by forced‐expressing four transcription factors OCT4, SOX2, KLF4 and C‐MYC,^3^ are regulated by core transcription factors OCT4 and SOX2, as well as NANOG^4^ to maintain their pluripotency. Both have the ability for self‐renewal with an unlimited proliferation and can be differentiated into almost all types of somatic cells.^1^ Therefore, hPSCs and their derivatives hold great potential for applications in the fields of biomedicine such as cell‐based therapies in regenerative medicine, and as a source of cells for use in disease modelling and drug discovery.^5^ However, before the applications of hPSCs and their derivatives can fulfil their potential, a significant challenge needs to be overcome with regards to culture scalability to produce clinically relevant numbers of cells (eg each treatment of myocardial infarction or diabetes requires 10^9^ cardiomyocytes or 10^9^ *β*‐cells, respectively).^6^

The conventional method of culturing hPSCs is on static two‐dimensional (2D) systems, which is only suitable for laboratory‐scale studies and cumbersome for scale‐up due to limited surface area. In addition, 2D culture systems fail to mimic the physiological environment in vivo or to provide sufficient signalling for stem cell proliferation with high efficiency and quality.^7^ For this reason, three‐dimensional (3D) systems have emerged as a promising strategy for large‐scale production of cells.

Currently, some progress has been made towards the development of suitable 3D culture systems for large‐scale expansion of hPSCs and their derivatives. One approach is to use microcarriers or microcapsules, which provide an advantage of increased surface area to volume ratio, providing more space for cell attachment and expansion.^8^, ^9^ However, this approach is characterized by its difficulty in cell detachment from microcarriers or microcapsules. Another strategy is microcarrier‐free suspension culture that yields a large number of cells. However, hPSCs tend to aggregate during culturing due to intercellular interactions. The control of aggregate size is crucial for maintaining the pluripotency of hPSCs and also for stable and efficient production of hPSCs. Excess aggregates hinder nutrients and oxygen from diffusing towards their interior, leading to hypoxia and central necrosis within cell aggregates and even affecting the pluripotency and differentiation potential.^10^ In general, physical and biochemical approaches are available to inhibit excess aggregation. One approach includes the regulation of agitation conditions in the bioreactor in an attempt to obtain size‐controlled aggregation.^11^, ^12^, ^13^ It is worth noting that an overly high shear stress produced by agitation could affect cell the viability and differentiation of hPSCs. Bauwens et al modulated aggregate size and shape by using microwells which functioned as physical barriers to limit cell movement. However, such a microwell‐based approach relies on the size and number of microwells, which is only applicable to laboratory‐scale studies.^14^ In addition to physical approaches, some chemical molecules or polymers have been used to control the aggregation of hiPSCs. Horiguchi et al demonstrated that KnockOut Serum Replacement (KSR) and lipid‐rich albumin are able to reproducibly prevent hiPSC aggregation without influencing pluripotency.^15^ Nath and his colleagues established a simple method for hiPSC aggregate break‐up by the addition of botulinum haemagglutinin to culture medium. They found that hiPSC aggregates broken up by HA showed a greater cell viability and expansion compared aggregates dissociated with enzymatic digestion; they reached a maximum cell density of 4.5 ± 0.2 × 10^6^ cells/mL.^16^ Dextran sulphate (DS), a polysulphated compound, has been used to prevent aggregation of cells in biopharmaceutical industry for decades. DS was recently reported to display aggregate control properties in hiPSCs, while not compromising the pluripotency of the cells.^17^, ^18^ Although the physical and biochemical approaches mentioned above could prevent cell aggregation and promote cell expansion efficiency to different degrees, none of these approaches were employed with the large numbers of cells that are necessary to meet clinical requirements. Poly(vinyl alcohol) (PVA) is a highly biocompatible and non‐toxic synthetic polymer that has a wide range of applications in the medical, cosmetic, food and pharmaceutical industries.^19^ In a recent study, Wilkinson et al developed a culture system for the long‐term ex vivo expansion of functional mouse haematopoietic stem cells (HSC) where serum albumin was replaced with PVA.^20^ They demonstrated that using this albumin‐free culture system led to a 236‐ to 899‐fold expansion of functional HSC over one month. To the best of our knowledge, the effect of PVA on hPSC expansion has not been investigated yet.

**FIGURE 1:**
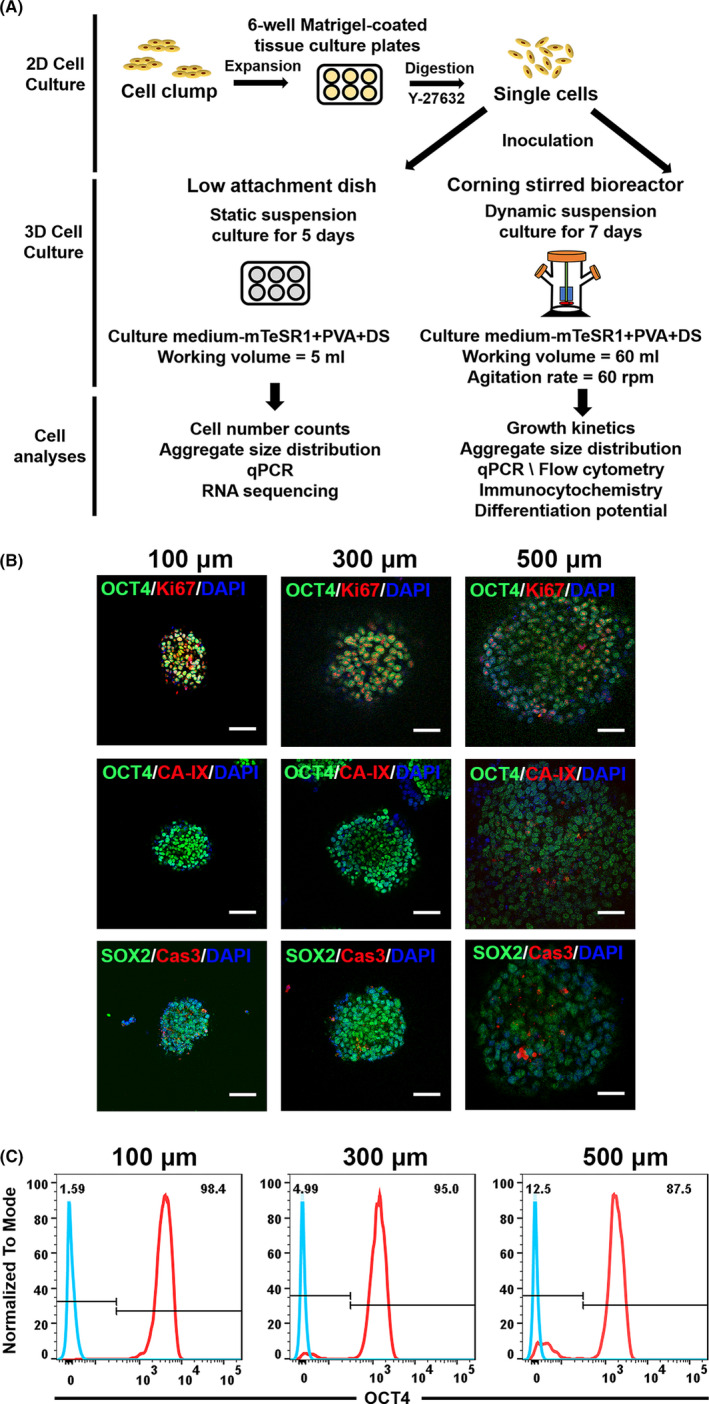
The effect of aggregate sizes on hPSCs in 3D suspension culture. (A), Schematic diagram of expansion of hPSC as aggregates in static or dynamic 3D suspension culture. (B), Immunofluorescence staining for markers OCT4, Ki67, CA‐IX, SOX2 and Caspase‐3 of different sizes of hPSC aggregate sections harvested from static suspension culture at day 5 post‐expansion. Scale bar = 100 μm. (C), Analysis of pluripotent markers OCT4 of different sizes of hPSC aggregates harvested from static suspension culture at day 5 post‐expansion with flow cytometry

In the present study, we developed a chemical‐based approach for ex vivo hPSC expansion by using a combination of PVA and DS. Our hypothesis was that PVA would promote the proliferation of hPSCs while DS would modulate cell aggregation. We further postulated that the combination of PVA and DS would not only yield a size‐controlled aggregate, but also significantly promote the growth of hPSCs in suspension culture. To test this hypothesis, we investigated the effect of DS and PVA as well as their combinations on aggregate formation, cellular proliferation and pluripotency of hPSCs in both static and dynamic suspension cultures (Figure 1A). Finally, we assessed the possible mechanisms and advantages of this approach.

## 2 MATERIALS AND METHODS

### 2.1 hPSCs culture

The hESC line, H9, was purchased from the WiCell Research Institute (Madison, WI, USA) under a Materials Transfer Agreement (No. 19‐W0512). The hiPSC line was provided by Dr Liangxue Lai's laboratory. For feeder‐free cultures, hPSCs were maintained in mTeSR1 (STEMCELL Technologies) medium on plates coated with hESC‐qualified Matrigel (Corning), and incubated at 37℃ in a humidified atmosphere with 5% CO_2_, and the medium was refreshed every day. After 5‐6 days culture, hPSC colonies were dissociated into single cells using Gentle Cell Dissociation Reagent (GCDR, STEMCELL Technologies). Cells were counted using a haemocytometer, and viable cells were identified by exclusion of trypan blue dye (Thermo Fisher). For subculture, cells were replated in a new culture dish at a viable cell density of 1 × 10^4^ cells per cm^2^.

### 2.2 Suspension expansion of hPSCs

For static suspension culture, hPSC colonies were dissociated with GCDR for 5‐7 minutes at 37℃ to obtain a single‐cell suspension; then cells were seeded into ultra‐low‐attachment 6‐well plates (Corning) at a cell density of 2 × 10^5^ cells per ml and cultured in mTeSR1 medium containing 10 μM Y‐27632 (STEMCELL Technologies), polyvinyl alcohol (PVA, MW = 31 000‐50 000, Hydrolysis: 87%‐89%, Sigma‐Aldrich) or Dextran Sulphate (DS, MW = 40 000, Sigma‐Aldrich), or the combination of PVA and DS. 60% of the culture medium was replaced with fresh medium without Y‐27632 each day. The PVA was supplemented during the entire period of the culture, and DS treatment was employed for only the first two days after inoculation. Cells were harvested by dissociation with TrypLE (Thermo Fisher) treatment at 37℃ for 15 minutes, and cell counts were performed by Trypan Blue exclusion (Thermo Fisher).

For dynamic suspension culture, hPSCs were cultured in disposable stirred bioreactors (Corning) with a maximum volume of 250 ml. Briefly, hPSC colonies were digested into single cells by using GCDR. Then, cells were seeded in a bioreactor at a density of 1 × 10^6^ cells per ml, and cultured in mTeSR1 media with Y‐27632 added until reaching the working volume. The medium was changed after 48 hours to mTeSR1 without Y‐27632, and 80% medium was refreshed every day. DS was supplemented only on day 1 at a concentration of 100 μg/mL while PVA was supplemented every day at a concentration of 1 mg/mL. Bioreactor cultures were maintained for 7 days, and the stirring speed was continuously maintained at 60 rpm/min.

Aggregate samples were taken on days 5‐7 and placed in a 6‐well culture dish (Corning) for photomicrographs using a Nikon D5100 camera attached to a Nikon Eclipse TS100 microscope. Three samples for each condition were taken and imaged. Image contrast and brightness were adjusted by ImageJ. ImageJ was used to measure the diameter of imaged cell aggregates.

### 2.3 Quantitative reverse transcription‐polymerase chain reaction (qRT‐PCR)

Total RNA was extracted from the cells using Universal RNA Extraction Kit (TaKaRa). Following quantification in a Nano Drop micro spectrophotometer (Thermo Fisher Scientific), 1 μg of RNA was converted to cDNA using the High‐Capacity PrimeScript^TM^ RT cDNA Reverse Transcriptase reagent Kit (TaKaRa). Reactions of cDNA generation were run in triplicate using PowerUp^TM^ SYBR^TM^ qPCR Green Master Mix (Thermo Fisher Scientific), and primers specific for GAPDH(F‐5′‐GAAGATGGTGATGGGATTTC‐3′, R‐5′‐GAAGGTGAAGGTCGGAGTC‐3′), OCT4(F‐5′‐AGGCAACCTGGAGAATTTGTTC‐3′, R‐5′‐CACACTCGGACCACATCCTTC‐3′), SOX2(F‐5′‐TACAGCATGTCCTACTCGCAG‐3′, R‐5′‐GAGGAAGAGGTAACCACAGGG‐3′) NANOG(F‐5′‐CAGGACAGCCCTGATTCTTCC‐3′, R‐5′‐TTTGCGACACTATTCTCTGCAGA‐3′) in a Quant Studio^TM^ 1 Real‐Time PCR System (Thermo Fisher Scientific). The cycle threshold (Ct) values for each condition were normalized to the corresponding expression of the housekeeping gene glyceraldehyde‐3‐phosphate dehydrogenase (GAPDH) to generate ΔCt. The RNA levels were calculated as 2^−ΔΔCt^.

### 2.4 FACS analysis

hPSC spheroids were dissociated into single cells by treatment with TrypLE™ Express (Gibco), and cells were fluorescently labelled by incubation with PE anti‐human TRA‐1‐81 antibody (Cat # 60065PE, STEMCELL Technologies), PE anti‐human SSEA‐4 antibody (Cat # 60062PE, STEMCELL Technologies) or PE mouse isotype‐controlled antibody (Cat # 556650, BD Pharmingen™). Fluorescence‐positive cells were then detected using a BD FACS Celesta flow cytometer.

### 2.5 Immunofluorescence of aggregates

The aggregate was collected and fixed by 4% paraformaldehyde (PFA) solution in PBS for 30 min at room temperature, washed in PBS and replaced with 30% sucrose in PBS at 4℃ overnight. The sucrose solution was discarded, and the aggregate placed in the cryomold following embedding with optimal cutting temperature. The 5‐mm thin sections were taken by CM1950 cryostat (Leica) and mounted onto glass slides for immunofluorescence staining. For aggregate intracellular staining, the frozen sections (5 µm) were air‐dried, fixed with 4% PFA for 10 minutes and permeabilized with 0.5% Triton X‐100 (Sigma‐Aldrich) for 20 minutes. Three washes with PBS were included between each step. Following washing, the samples were incubated in blocking buffer containing goat serum for 30‐60 minutes at room temperature, then incubated in PBS containing primary antibodies (anti‐OCT4 Rabbit IgG, 1:200, Cat # 2750; anti‐SOX2 mouse IgG, 1:400, Cat # 4900; anti‐NANOG Rabbit IgG, 1:200, Cat # 4903; anti‐Ki67 mouse IgG, 1:800, Cat # 9449; anti‐Cleaved Caspase‐3 Rabbit IgG, 1:800, Cat # 9664; Cell Signaling Technology; anti‐Carbonic anhydrase IX antibody [GT12] mouse IgG, 1:800, Cat # 70020, GeneTex) overnight at 4℃ followed by rewarming to room temperature and incubated in PBS containing secondary antibodies (Alexa Fluor 488‐conjugated goat anti‐mouse IgG, 1:800, Cat # 4408S; Alexa Fluor 488‐conjugated goat anti‐Rabbit IgG, 1:800, Cat # 4412S; Alexa Fluor 594‐conjugated goat anti‐Rabbit IgG, 1:800, Cat # 8889S; Alexa Fluor 594‐conjugated goat anti‐mouse IgG, 1:800, Cat # 8890S; Cell Signaling Technology) for 1 hour in the dark at room temperature. Counter staining was performed with DAPI (Cell Signaling Technology) for 5 minutes, and the fluorescence signal was imaged on the single photon confocal microscopy (Ti‐E A1, Nikon).

### 2.6 Embryoid body formation assay

The pluripotent capability and differentiation potential of hPSCs were evaluated in vitro through the formation of the embryoid body (EB), which directly differentiated into all three germ layers: endoderm, mesoderm and ectoderm lineages in EB medium.

Briefly, hPSCs from the Corning stirred bioreactor were replated on 6‐well ultra‐low‐attachment tissue culture plates (Corning) in mTeSR1 supplemented with Y‐27632. After 24 hours, the culture medium was changed to EB differentiation medium, composed of Knock Out (KO)‐Dulbecco's Modified Eagle's Medium, 20% foetal bovine serum (FBS), 1% non‐essential amino acids, 1mmol/L L‐glutamine and 1% (V/V) penicillin/streptomycin (all from Thermo Fisher Scientific). The EB differentiation medium was refreshed every 2 days. After two weeks, the EBs were stained for SOX17 (anti‐SOX17 mouse IgG, 1:800, Cat # ab84990, Abcam), Brachyury (anti‐Brachyury Rabbit IgG, 1:800, Cat # 81694S, CST) and GFAP (anti‐GFAP Chicken IgG, 1:800, Cat # ab4674, Abcam) and incubated in PBS containing secondary antibodies (Alexa Fluor 488‐conjugated goat anti‐Rabbit IgG, 1:800, Cat # 4412S; Alexa Fluor 594‐conjugated goat anti‐mouse IgG, 1:800, Cat # 8890S, Cell Signaling Technology; Alexa Fluor 647‐conjugated goat anti‐Chicken IgG, 1:800, Cat # ab150171, Abcam) and observed under single photon confocal microscopy (Ti‐E A1, Nikon). Then, the EBs were dissociated into small cell clumps using 0.05% Trypsin‐EDTA (Thermo Fisher Scientific) and transferred onto Matrigel‐coated plates for the differentiation. EB medium was changed every 2 days for one week, and then the differentiated cells were stained with SOX17, Brachyury and GFAP, and observed under single photon confocal microscopy (Ti‐E A1, Nikon).

### 2.7 Teratoma formation assay

All animal procedures were approved by the Animal Ethics Committee of South China University of Technology. Immunodeficient NDG mice were purchased from Beijing Biocytogen, China, and housed under specific pathogen‐free conditions with a 12‐hours light/dark cycle.

hPSC spheroids were dissociated into single cells by treatment with TrypLE™ Express. 1 × 10^6^ hPSCs were mixed with Matrigel and subcutaneously injected into NDG mice. The mice were maintained under specific pathogen‐free (SPF) conditions and fed with a sterilized pelleted diet and water. After 4‐8 weeks of injection, the mice were sacrificed, and teratomas were dissected, fixed in 4% paraformaldehyde and embedded in paraffin. The paraffin block was sectioned to a thickness of 10 µm, and tissue sections were stained with haematoxylin and eosin.

### 2.8 Cell Counting Kit‐8 assay

Cell viability was assessed using the Cell Counting Kit (CCK)‐8 assay (Sigma‐Aldrich) according to the manufacturer's instructions. Briefly, hPSCs (2 × 10^3^ cells per well) were seeded in 96‐well plates with 100 μl of medium. After various cell treatments, 10 µl of CCK‐8 solution was added to each well and incubated in a humidified incubator for 2 hours at 37℃ and 5% CO_2_. The optical density values were measured at 450 nm using a microplate reader (Cytation 5, Bio‐Tek). Wells without cells served as blank controls. Each experiment was performed in triplicate.

### 2.9 Glucose and lactate analysis

Culture supernatants were collected every day prior to and following medium exchange, and centrifuged at 360 g for 10 minutes to remove dead cells and debris. The cell‐free supernatants were analysed using an Automatic Biochemistry Analyser (3100, Hitachi) for concentrations of glucose and lactate. The apparent yield of lactate from glucose was calculated for each day as

$$Y_{\text{Lac}/\text{Glc}}=\Delta \text{Lac}/\Delta \text{Glc}.$$

with ΔLac as the production of lactate and ΔGlc as the consumption of glucose during a given day of culture.

### 2.10 Transcriptome sequencing (mRNA‐seq) and data analysis

Total RNAs from H9 in static suspension culture supplemented with 100 μg/mL DS, 1 mg/mL PVA or their combinations were respectively extracted using Trizol according to the manufacturer's instructions (TaKaRa). H9 in static suspension culture without supplementation was set as control. RNA sequencing libraries were generated using NEBNext® Ultra™ RNA Library Prep Kit for Illumina® (NEB, USA). Sequencing was performed by Novogene (China). Sequencing was performed on an Illumina HiSeq X‐Tensequencer with 150 bp paired‐end sequencing reaction. The bulk RNA‐Seq data for hPSCs were downloaded from the GEO database. The reads were mapped to the human reference genome using HISAT2. Reads Counts for each gene in each sample were counted by featureCounts, and FPKM (Fragments Per Kilobase Millon Mapped Reads) and then were calculated to estimate the expression level of genes in each sample. Differentially expressed genes (DEGs) were analysed by DESeq2 using counts. Genes with *P* value ≤.05 and |log2 Fold Change| ≥1.5 (PVA group, *P* value ≤.05 and |log2 Fold Change| ≥0.4) are identified as DEGs. Heatmap generation was performed with the R package. TBtools software and DAVID database were used to test the statistical enrichment of differential expression genes in KEGG pathways and Gene Ontology. Original data were uploaded to the Gene Expression Omnibus database (accession number PRJNA699756).

### 2.11 Statistical analysis

Data are expressed as the mean ± standard deviation (n = 3). Statistical analysis was performed using GraphPad Prism 6 (GraphPad Software, USA), and statistical significance was determined by Student's *t* Tests. Differences were considered statistically significant where a *P* value was <.05.

## 3 RESULTS AND DISCUSSION

In recent years, there has been a strong drive towards translating basic research of hPSCs into industries and clinics. One of the key elements for successful translational applications is the ability to produce hPSCs in a scalable and quality‐controlled manner. As such, a variety of approaches have been taken by researchers for large‐scale expansion of hPSCs.^8^, ^9^ Aggregate‐based expansion methods hold great promise for scalable expansion of hPSCs due to the relative simplicity and reduced processing steps required. Nevertheless, most aggregate expansion methods to date generate heterogenous and excess aggregate sizes which have negative influence on cellular viability and differentiation potential.

### 3.1 Effect of aggregate sizes on hPSC quality in suspension culture

Aggregate size control has been recognized as one of critical parameters for mass production of hPSCs using aggregate‐based suspension culture systems.^21^ However, few studies have described how the aggregate sizes affect hPSC quality in suspension culture. To this end, we collected hPSC aggregates of different sizes (100, 300 and 500 μm in diameter) from static suspension culture, performed frozen sections (5 µm) and immunofluorescence staining analysis (Figure 1B). We evaluated the frequency of cell division across aggregates using Ki‐67 as a marker of proliferation. Proliferating cells were common throughout hPSC aggregates regardless of sizes. Cells in the aggregates with diameters around 100 and 300 μm showed uniform and comparable OCT4 expression. In contrast, cells in aggregates of 500 μm in diameter have reduced pluripotent marker OCT4 expression, indicating a loss of pluripotency. These results were further confirmed by FACS analysis (Figure 1C). The OCT4 expression was observed in 98.4% and 95% of cells in the aggregates with diameters around 100 μm and 300 μm, respectively, while the expression value dropped down to 87.5% for the cells in aggregates of 500 μm in diameters. Next, we evaluated the presence of senescent cells within aggregates using apoptotic indicator cleaved caspase‐3. Senescent cells were present at a moderate frequency within small aggregates (100‐300 μm in diameter) and were found to be prominent within large cell aggregates (500 μm in diameter). To investigate the presence of hypoxia throughout aggregates, we stained hPSC aggregates with carbonic anhydrase IX (CA‐IX). Our data demonstrated that CA‐IX‐positive cells were only densely located near the centre of aggregates with a diameter of 500 μm, indicating the lack of oxygen at the centre of aggregates. Our results are in agreement with a previous study by Wu et al They described that if the aggregate diameter was greater than 300 μm which is the diffusion limit of essential factors, the cells inside the centre of the aggregate would suffer from a lack of oxygen and nutrient transport, and thus damage cell pluripotency.^22^

Therefore, it appears that it is necessary to control the size of the aggregates by physical methods such as controlling the stirring rate, and chemical methods such as adding small molecules to obtain uniform cell aggregate products with sufficient exposure to oxygen, nutrients and media growth factors.

### 3.2 Effect of DS on hPSC static suspension culture

**FIGURE 2:**
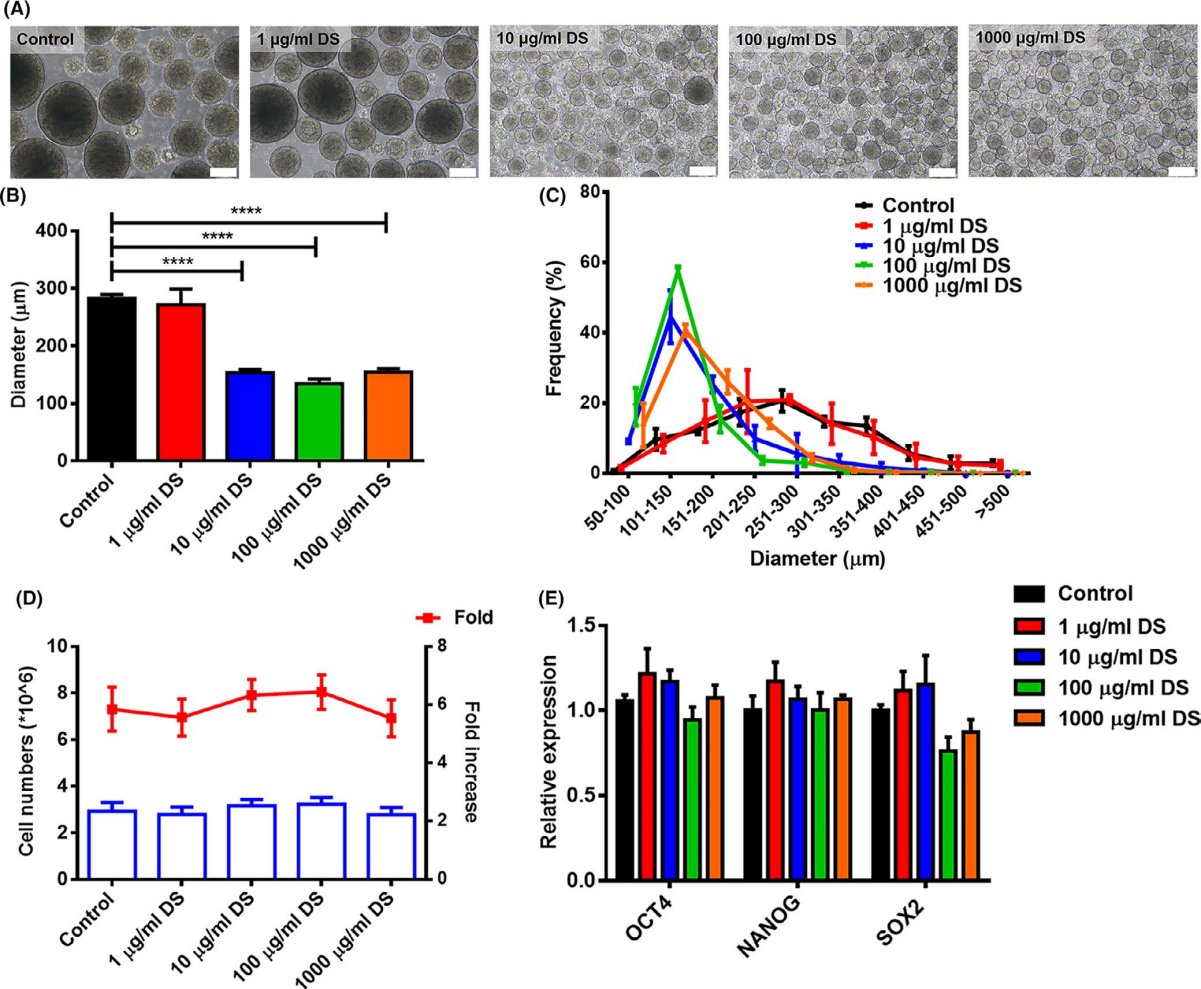
The effect of DS on hiPSCs in static suspension culture. (A), Representative images of hiPSC aggregates on day 5 after treatment with various DS concentrations. Scale bar = 200 μm. (B), Comparison of average diameter of hiPSC aggregates on day 5. (C), Diameter distribution of hiPSC aggregates treated with various concentrations of DS. (D), Comparison of cell yield after 5 d of culture. (E), Gene expression analysis by qPCR for pluripotent genes, OCT4, NANOG and SOX2 after 5 d of culture. ***P < .001

DS is a well‐characterized polysulphate compound that has been used to prevent cell aggregation in biopharmaceuticals.^23^, ^24^, ^25^ Recent work by Lipsitz YY et al compared the effect of DS molecular weight (4,000 kDA, 15,000 kDA and 40,000 kDA) on the properties of hPSC aggregation and suggested that the addition of DS to culture medium resulted in the formation of aggregates with significantly reduced diameters in a dose‐dependent fashion.^17^ Particularly, 40 000 kDA DS showed the best performance for 3D culture of hPSCs. Therefore, we chose 40 000 kDA DS in the present study. To evaluate the effect of DS on hPSC aggregation, we first varied the concentration of DS in small scale static suspension culture. The addition of DS significantly changed hiPSC aggregation properties in all tested concentrations except 1 µg/mL (Figure 2A‐C). In the absence of DS, hiPSC aggregates were heterogeneous with an average aggregate size of 286 ± 116 μm. As DS concentration increased from 1 µg/mL to 10 µg/mL, the hiPSC aggregates became more homogeneous, and the average aggregate size was significantly reduced to 154 ± 54 μm from 257 ± 99 μm (Figure 2B, C). When the DS concentration further increased to 1000 µg/mL, there was no significant change in aggregate diameter (150 ± 54 μm) and size distribution (Figure 2B, C). Treatment with 100 µg/mL DS resulted in the lowest aggregate size (134 ± 51 μm) among the concentration investigated (Figure 2B, C). Anti‐apoptotic activity and cell surface charge modulation may account for the aggregation control effects of DS on hPSCs.^24^ Although the DS treatment could lead to uniform and small aggregate formation, the effect of DS on the proliferation of hPSCs is controversial. In our study, we did not observe DS treatment to promote cellular proliferation, as evidenced by the total cell density (2.99 ± 0.23 × 10^6^ cells/mL) which is not significantly higher than that of control group (2.92 ± 0.32 × 10^6^ cells/mL) (Figure 2D). Our results are consistent with the findings by Lipsitz and his colleagues.^17^ However, in another study, Nogueira et al reported that the use of mTeSR1 or mTeSR3D media with 100 µg/mL DS led to a 97 or 106% increase in total cell numbers respectively vs the medias without DS.^18^ qRT‐PCR results demonstrated that in all the investigated concentrations of DS, hiPSCs expressed high levels of pluripotent marker genes comparable to that of control culture (Figure 2E). These findings suggest that DS treatment enables the formation of uniform aggregates without losing pluripotency. Previous studies also demonstrated that adding DS during the cultivation process could control the aggregation characteristics of hPSCs without losing pluripotency.^17^

### 3.3 Effect of PVA on hPSC static suspension culture

**FIGURE 3:**
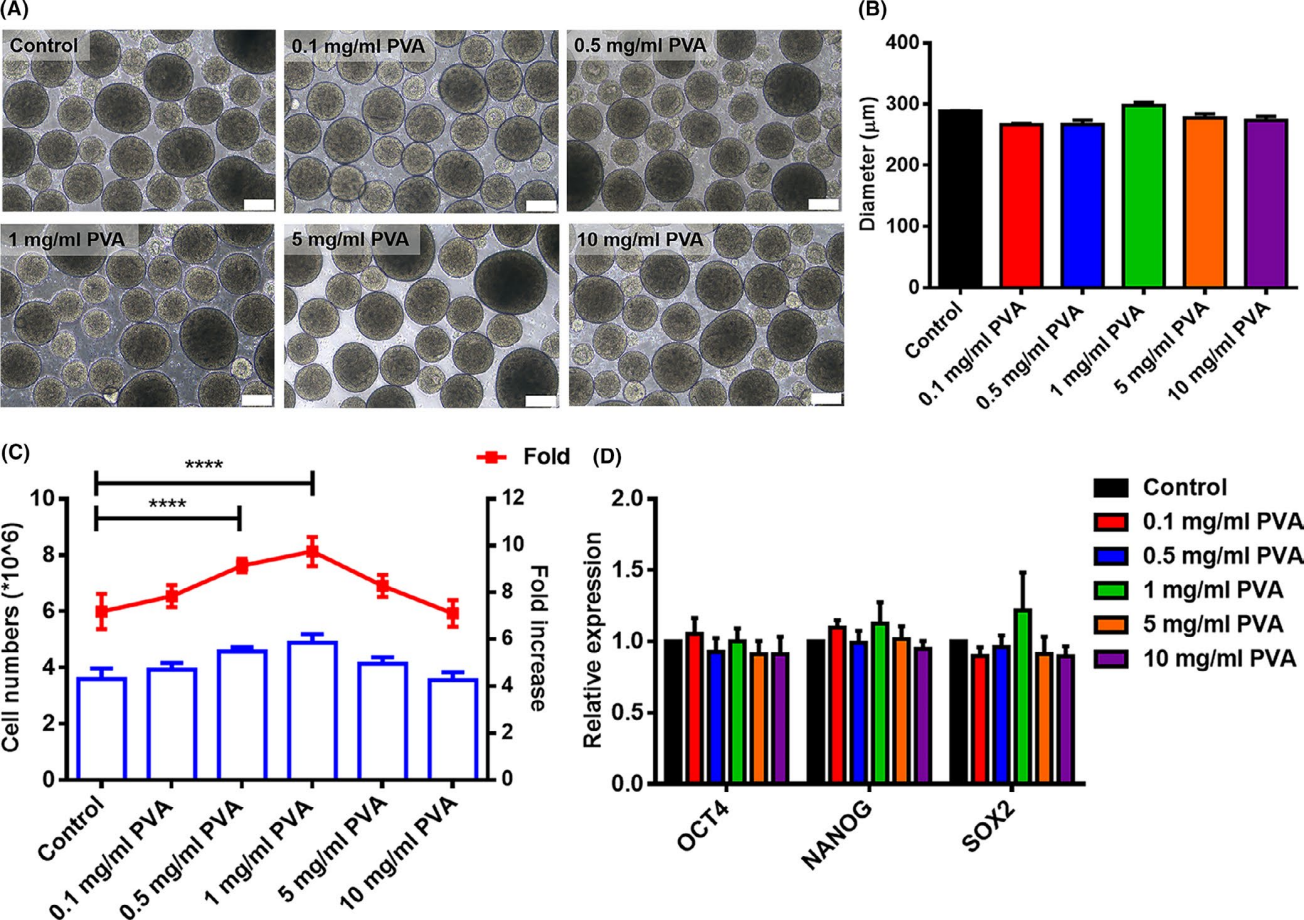
The effect of PVA on hiPSCs in static suspension culture. (A), Representative images of hiPSC aggregates on day 5 after treatment with various PVA concentrations. Scale bar = 200 μm. (B), Comparison of average diameter of hiPSC aggregates on day 5. (C), Comparison of cell yield after 5 days of culture. (D), Gene expression analysis by qPCR for pluripotent genes, OCT4, NANOG and SOX2 after 5 d of culture. ***P < .001

PVA, a common and cheap synthetic polymer, has been widely used in biomedical applications for its nontoxicity, non‐carcinogenic and bioadhesive properties.^26^ To investigated the effect of PVA treatment on hPSC expansion, hiPSCs were plated as single cells in low‐attachment plates under static condition, and treated with a variety of PVA concentrations ranging from 0.1 to 10 mg/mL over a five‐day culture period, we did not observe any change in aggregate uniformity or aggregate size relative to the untreated condition (Figure 3A, B). Supplementation with 1 mg/mL PVA resulted in the highest cell density (4.88 ± 0.29 × 10^6^ cells/mL) representing a 9.76‐fold expansion, which was 1.36 times higher than that of control culture (3.59 ± 0.35 × 10^6^ cells/mL) (Figure 3C). A similar finding was observed for hiPSCs in adherent 2D cultures (Figure S1), and hiPSCs maintained a typical colony morphology after PVA treatment (Figure S1A). Compared with control culture, a significant increase in total cell numbers was observed in treatment with 0.5 mg/mL and 1 mg/mL PVA, which was further confirmed by cell viability analysis using CCK‐8 assay (Figure S1B, C). qRT‐PCR analysis demonstrated that hiPSCs aggregates treated with PVA had similar expression profiles of pluripotent marker genes, including OCT4, NANOG and SOX2 (Figure 3D). These findings indicate that PVA treatment could significantly promote hPSC proliferation without causing loss of pluripotency.

### 3.4 Effect of the combination of PVA with DS on hPSC suspension culture

Next, we determined whether the combined addition of DS and PVA could improve cellular proliferation while reducing aggregate size and size variability. To obtain an optimal formulation, we first investigated the effects of DS and PVA in different concentrations and their combinations on hiPSCs in static suspension culture (Figures S2 and S3). Our results showed that the combination of 1 mg/mL PVA and 100 μg/mL DS yielded the best outcome.

**FIGURE 4:**
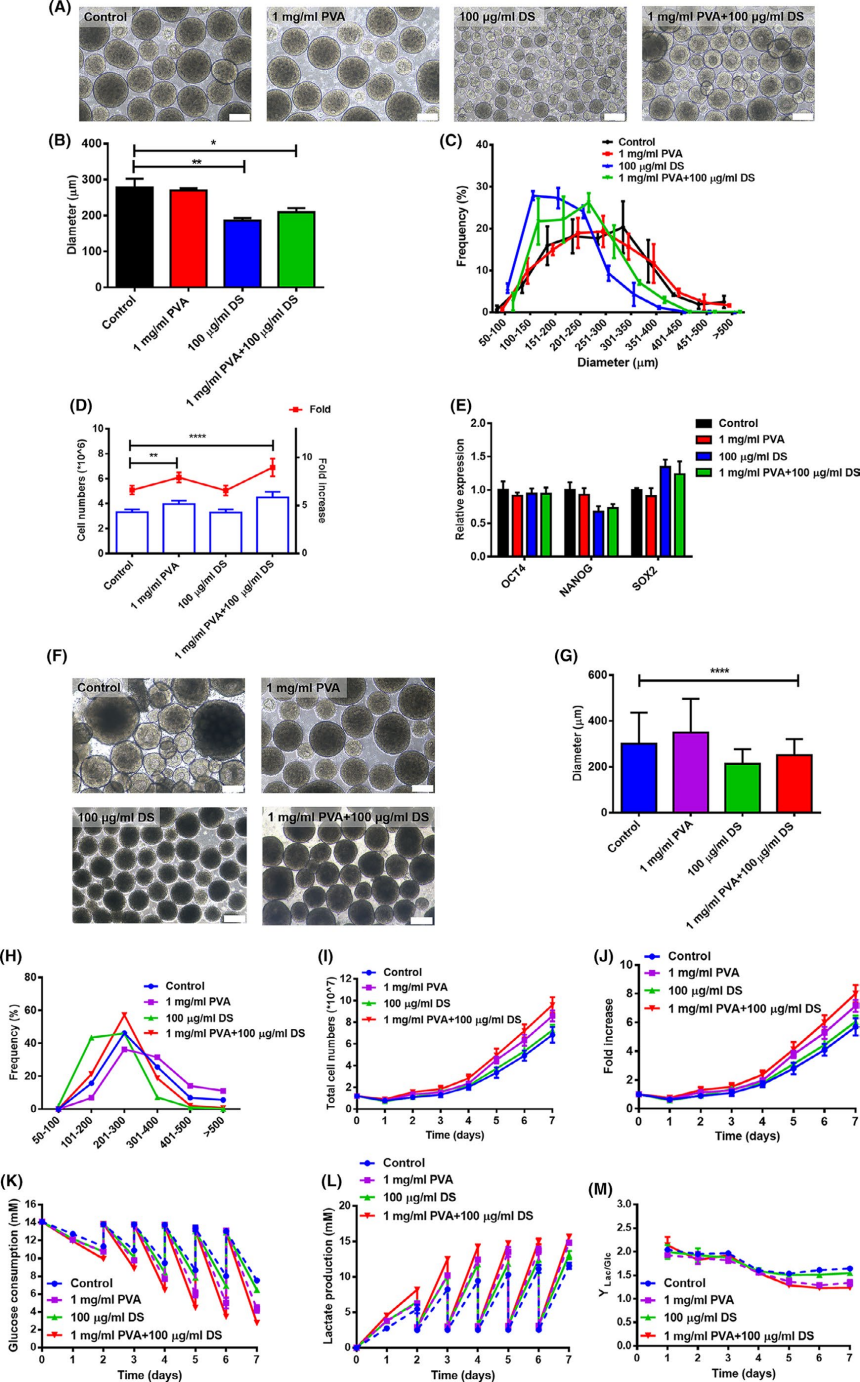
The effect of the combination of PVA and DS on hiPSCs in static or dynamic suspension culture. (A), Representative images of hiPSC aggregates on day 5 in static suspension culture. Scale bar = 200 μm. (B), Comparison of average diameter of hiPSC aggregates on day 5 in static suspension culture. (C), Diameter distribution of hiPSC aggregates on day 5 in static suspension culture. (D), Comparison of cell yield after 5 d in static suspension culture. (E), Gene expression analysis by qPCR for pluripotent genes, OCT4, NANOG and SOX2 after 5 d in static suspension culture. (F), Representative images of hiPSC aggregates on day 7 in dynamic suspension culture, scale bars = 200 μm. (G), Average diameter of hiPSC aggregates on day 7 in dynamic suspension culture. (H), Diameter distribution of hiPSC aggregates on day 7 in dynamic suspension culture. (I), Growth kinetics of cell numbers of dynamic suspension culture. (J), Growth kinetics of cell fold increase of dynamic suspension culture. (K), Glucose concentration analysis of cell culture supernatant. (L), Lactate production analysis of cell culture supernatant. (M), Yield of lactate from glucose of dynamic suspension culture. *P < .05; **P < .01; ***P < .001

As shown in Figure 4A‐C, after 5 days in static suspension culture, hiPSCs treated with 1 mg/mL PVA and 100 µg/mL DS had an average aggregate size of 207 ± 67 μm which is slightly larger than that of hiPSCs treated with 100 µg/mL DS alone (179 ± 60 μm), but significantly smaller than that of control culture (293 ± 99 μm). In terms of cell yield, hiPSCs treated with 1 mg/mL PVA and 100 µg/mL DS showed a 36% increase in the numbers of cells vs the control culture (Figure 4D).

Similar findings were also observed within the H9 cell line (Figure S4). H9 treated with 1 mg/mL PVA and 100 µg/mL DS had a more uniform, smaller aggregate morphology with an average aggregate size of 191 ± 38 μm (Figure S4A, B). Compared with control culture, H9 treated with 1 mg/mL PVA and 100 µg/mL DS showed a 53% increase in total cell numbers (Figure S4C). Additionally, the expressions of pluripotent genes (OCT4, NANOG and SOX2) (Figure 4E) and flow cytometry analyses (Figure S4D, E) suggest that it is an appropriate cultivation process without losing pluripotency.

We then employed this approach in scalable stirred bioreactors for hiPSC expansion where the combination of PVA with DS not only led to form uniform aggregate sizes, but also significantly promoted cell yields (Figure 4F‐J). After 7 days of stirred suspension culture, untreated hiPSCs displayed large, heterogeneous aggregates with an average aggregate size of 301 ± 136 μm. In contrast, hiPSCs treated with the combination of PVA and DS had a more controlled aggregate size of 250 ± 69 μm (Figure 4G). In addition, hiPSCs treated with combination of PVA and DS had a cell density of 1.6 ± 0.33 × 10^6^ cells/mL (8‐fold increase) on the 7th days post inoculation, representing a 40.35% increase in the number of cells vs the control culture (Figure 4I, J). Our strategy may be superior to the recent work by Manstein et al They attempted to expand hPSCs in a stirring‐controlled bioreactor by addition of Pluronic F68 as shear protectant.^27^ Despite stirring‐controlled reduction in aggregate diameters, the final cell yield did not increase but rather dropped.

To evaluate the effect of PVA and DS on cellular metabolite consumption and production, the concentrations of glucose and lactate were monitored during the cultures, as shown in Figure 4K‐M. Considerable glucose depletion was found for every media, and a higher cell‐specific glucose consumption was observed in response to the PVA and PVA + DS treatment (Figure 4K). Regardless of the addition of PVA and DS, glucose consumption was accompanied by a corresponding accumulation of lactate throughout the cultures, and lactate was built‐up until nearly 16 mm in the PVA + DS treatment (Figure 4L). We then further calculated the yield of lactate from glucose (Figure 4M). For Control, following a yield of lactate from glucose of ~2.0 at day 1, it stabilized between 1.6 and 1.7 towards the end of culture. For PVA + DS, this yield was maintained at ~2.0 for the initial days of culture, reaching ~1.3 at the end of culture. These results indicate the metabolism, most likely, have being predominantly glycolysis, oxidation‐reduction process and oxidative phosphorylation, in particular with PVA and PVA + DS group.

**FIGURE 5:**
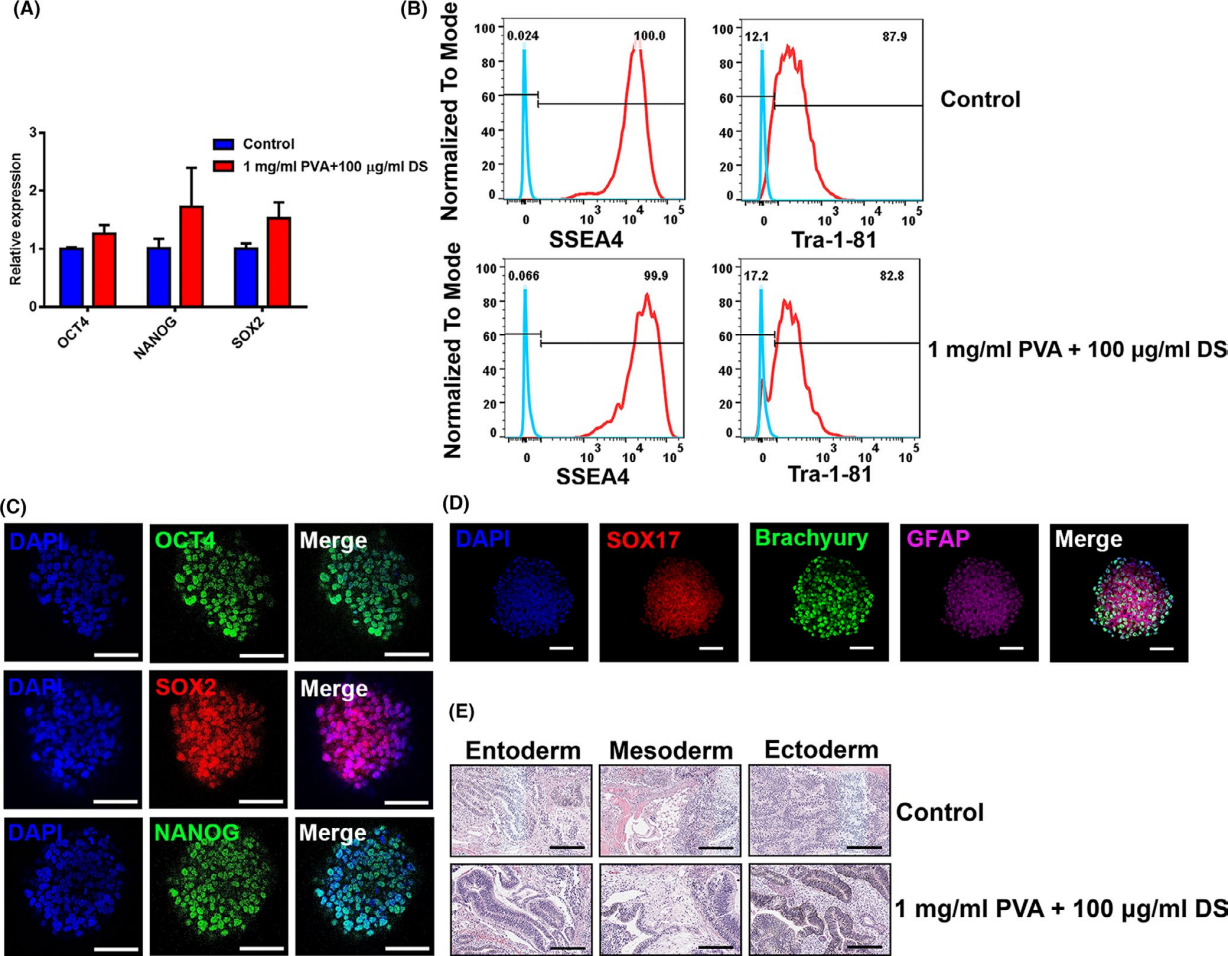
Characterization of the pluripotency and differentiation capacity of hiPSCs after 7 d in dynamic suspension culture supplemented with 1 mg/mL PVA and 100 μg/mL DS. (A) Gene expression analysis of hiPSCs by qPCR for pluripotent genes, OCT4, NANOG and SOX2. (B), Analysis of pluripotent markers with flow cytometry. (C), Immunofluorescence analysis of hiPSCs for pluripotent markers, OCT4, SOX2 and NANOG after treatment with 1 mg/mL PVA and 100 μg/mL DS. Scale bars = 100 μm. (D), Immunofluorescence analysis of hiPSCs for three germ layer markers, GFAP (ectoderm), Brachyury (mesoderm) and SOX17 (endoderm). Scale bars = 50 μm. (E), Section of teratomas from hiPSCs with or without treatment with 1 mg/mL PVA and 100 μg/mL DS. Scale bars = 200 μm

Quantification of pluripotent marker expression was performed using qRT‐PCR analysis, flow cytometry and immunofluorescence. There was no significant difference in the expressions of pluripotent genes (OCT4, NANOG and SOX2) between treated and untreated hiPSCs (Figure 5A). Flow cytometry analyses revealed that the expressions of pluripotent markers SSEA‐4 and TRA‐1‐81 in treated hiPSCs were comparable to those in control culture (Figure 5B). Finally, we examined the pluripotency of treated hiPSCs at day 7 by immunofluorescence and found that these hiPSCs aggregates co‐expressed OCT4, SOX2 and NANOG (Figure 5C). These findings demonstrated that the combination of DS and PVA enabled the formation of uniform aggregates without causing a loss of pluripotency in the dynamic suspension culture.

Harvested hiPSC aggregates after 7 days of culture in stirred suspension were then dissociated into single cells with GCDR and replated on Matrigel‐coated 2D tissue culture plates. These cells were able to successfully return to static culture conditions and then form hiPSCs colonies (Figure S5A). Representative images show strong expression of surface pluripotent markers, SSEA‐4 and TRA‐1‐81 (Figure S5B).

The differentiation capability of hiPSCs from dynamic suspension culture was also investigated. Embryoid bodies (EBs) generated from hiPSCs were able to spontaneously differentiate into the three germ layers, as differentiated cells were stained positive for specific markers of three germ layers, GFAP (ectoderm) and Brachyury (mesoderm) as well as SOX17 (endoderm; Figure 5D), and then the EBs were transferred onto Matrigel‐coated plates. These cells were also stained by immunofluorescence for germ layer markers GFAP, Brachyury and SOX17 (Figure S5C).

A teratoma assay was utilized to evaluate the capacity for differentiation in vivo. hiPSCs from suspension culture in spinner flasks were injected into immune‐deficient mice and were found to form teratomas containing tissues from three germ layers; for example, adipose tissue (mesoderm), intestinal epithelium (endoderm), as well as neuroepithelium and pigment epithelial (ectoderm) (Figure 5E and Figure S5D, E).

### 3.5 mRNA‐seq analyses

To investigate the mechanisms underlying the aggregation control and cell yield increase by DS and PVA respectively, we performed mRNA‐seq analyses. Heatmaps comparing differentially expressed genes (DEGs) induced in hPSCs in response to DS, PVA and PVA plus DS treatment are shown in Figure S6. Results demonstrated that significant changes occurred in the transcriptomic profile when treated with the chemical compounds investigated.

**FIGURE 6:**
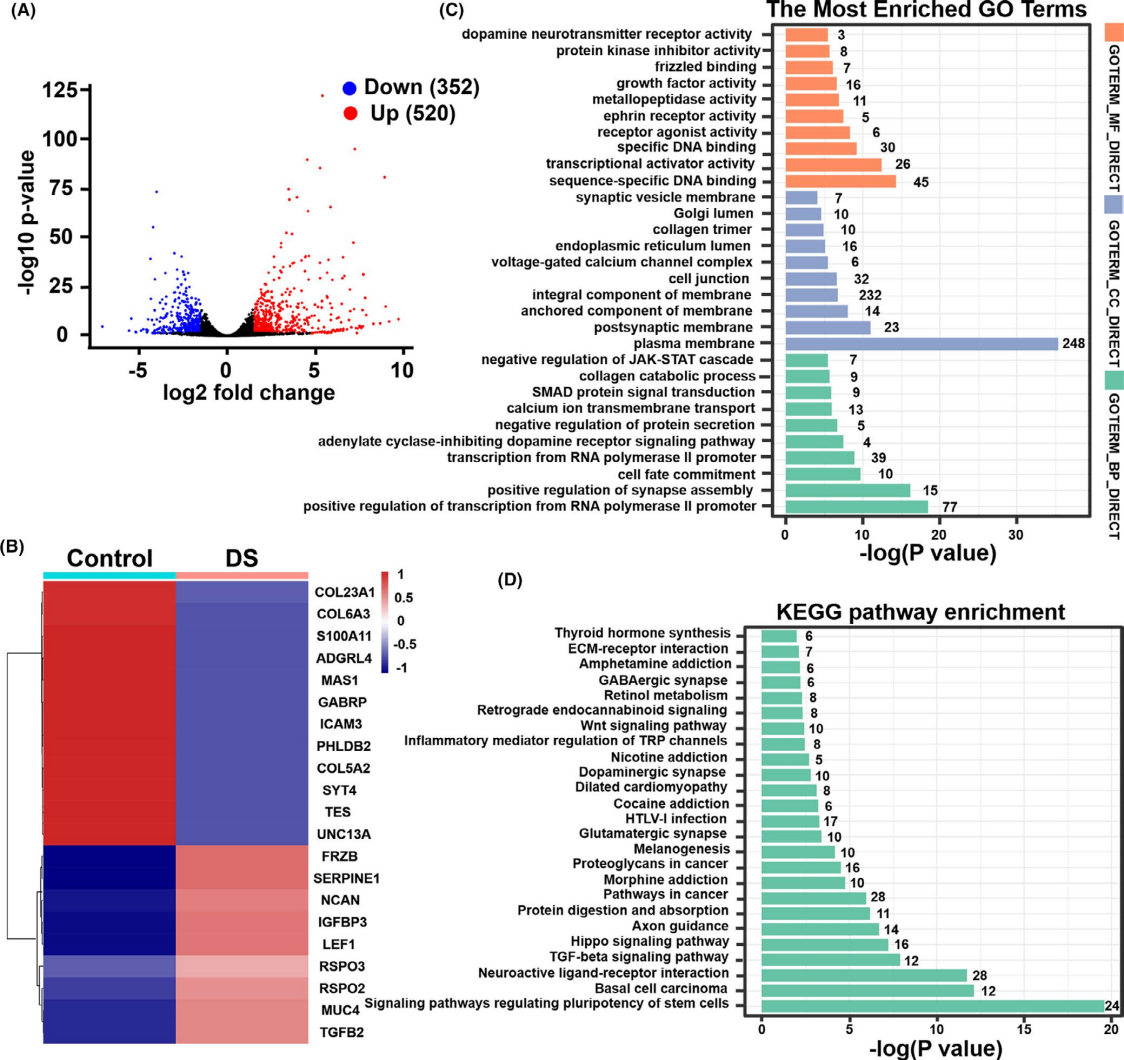
Transcriptome profiles of DS on hPSCs in static suspension culture. (A), Volcano map of DEGs of hPSCs in DS vs Control. Red dots indicate significantly upregulated DEGs; blue dots indicate significantly downregulated DEGs; and grey dots indicate no significance. (B), Heatmap of DEGs of hPSCs, Control vs DS. (C), GO terms of DS groups. (D), KEGG pathway enrichment of hPSCs, Control vs DS. Black number is the number of enriched genes

The volcano maps show that there are 352 downregulated and 520 upregulated differentially expressed genes (DEGs) between the Control and DS group (Figure 6A). Heatmap of DEGs between Control and DS treatment was presented in Figure 6B. We found that some genes showed higher expression levels in the DS group than those in the control group. These genes involve the integral component of membrane (NCAN,^28^ MUC4^29^), plasma membrane (SERPINE1^30^, ^31^), protein phosphorylation (IGFBP3,^32^, ^33^ TGF*β*2^34^) and Wnt signalling pathway (LEF1,^35^ RSPO2 and RSPO3,^36^ FRZB^37^). The function of these genes has been reported to inhibit cell adhesion. In addition, we found that some genes showed lower expression levels in the DS group than in the control group. These genes involve the plasma membrane and integral component of membrane (ICAM3^38^), collagen trimer (COL^39^), cadherin binding involved in cell‐cell adhesion^40^ (PHLDB2, TES and S100A11), G‐protein‐coupled receptor activity^41^ (ADGRL4, MAS1) and cell junction (UNC13A, SYT4 and GABRP). The function of these genes has been reported to improve cell adhesion. Many studies have shown that cadherin binding and G‐protein‐coupled receptor play an important role in cell adhesion. Azarin et al reported that initial PSC aggregation was mediated by cadherin‐cadherin interactions.^40^ The size of PSC aggregates regulated the expression level of E‐cadherin, which modulates Wnt signalling.^21^ Adhesion G‐protein‐coupled receptors (AGPCRs) are a large family of transmembrane proteins that function primarily through cell‐cell and cell‐extracellular matrix (ECM) interactions.^41^ The gene ontology (GO) analysis showed that upregulated and downregulated DEGs in the DS group were mainly related to plasma membrane, integral component of membrane, cell junction, collagen trimer, growth factor activity, cell fate commitment and calcium ion transmembrane transport, respectively (Figure 6C). The up‐ and downregulated DEGs between DS treatment and control groups were enriched in 25 pathways by KEGG analysis (Figure 6D). The TGF‐beta signalling pathway, Hippo signalling pathway and Wnt signalling pathway may be the most important pathways. Taken together, our results indicate that DS treatment might reduce the adhesion among hPSC aggregates through affecting expression of genes related to cell adhesion.

**FIGURE 7:**
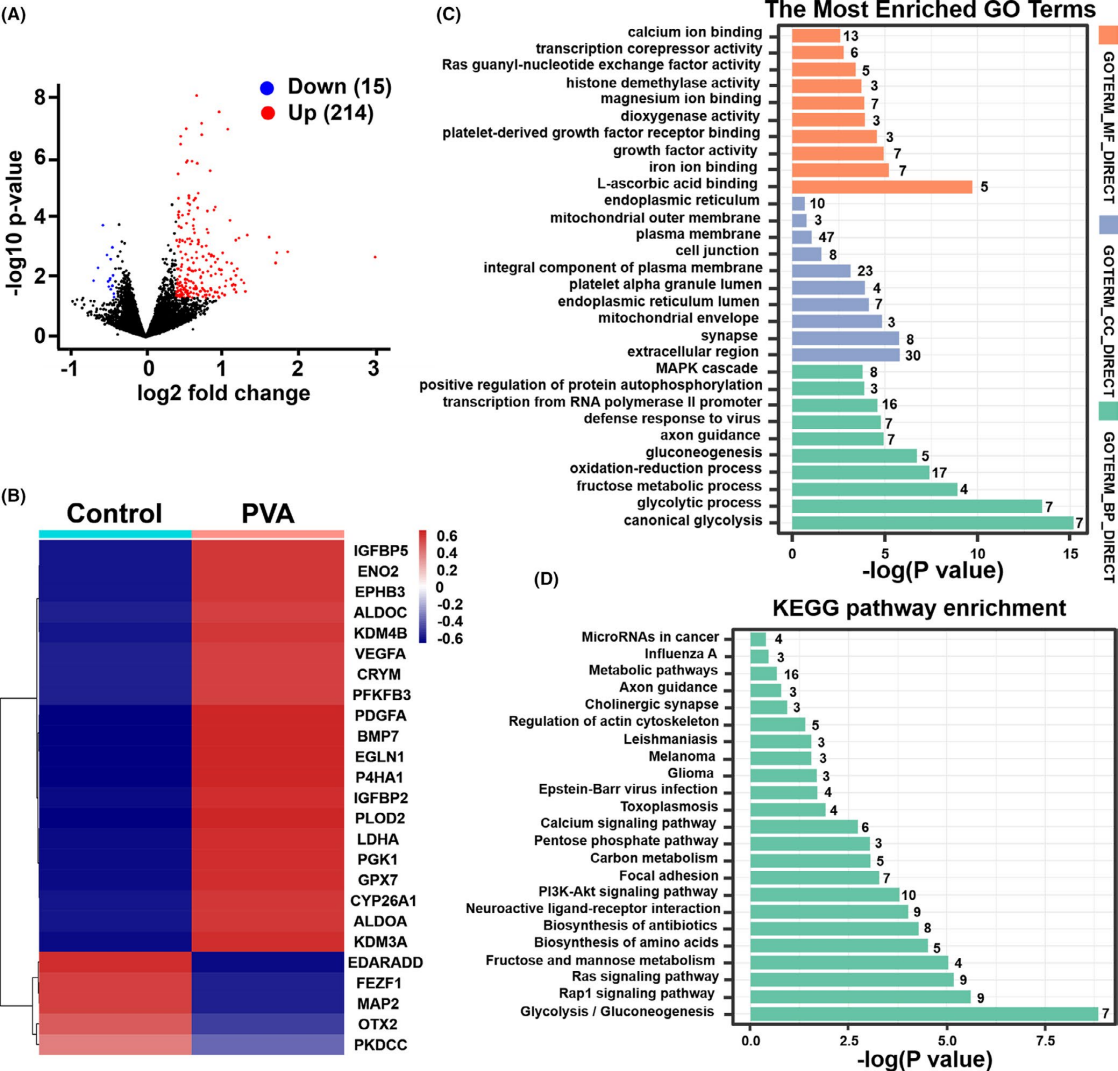
Transcriptome profiles of PVA on hPSCs in static suspension culture. (A), Volcano map of DEGs of hPSCs in PVA vs Control. Red dots indicate significantly upregulated DEGs; blue dots indicate significantly downregulated DEGs; and grey dots indicate no significance. (B), Heatmap of DEGs of hPSCs among Control and PVA. (C), GO terms of PVA groups. (D), KEGG pathway enrichment of hPSCs among Control and PVA. Black number is the number of enriched genes

Next, we compared gene expression patterns between PVA treatment and control groups. There were 15 downregulated and 214 upregulated DEGs between the PVA and Control groups (Figure 7A). Heatmap of DEGs between Control and PVA treatment is shown in Figure 7B. The higher expression genes involved in canonical glycolysis process (PGK1, PFKFB3, ALDOC, ALDOA, ENO2, LDHA),oxidation reduction process (CYP26A1, PLOD2, P4HA1, EGLN1, KDM4B, LDHA, GPX7, KDM3A, CRYM), fructose metabolic process (PFKFB3, ALDOA, ALDOC), growth factor activity (PDGFA, VEGFA, BMP7), ATP binding (PGK1, EPHB3, PFKFB3, PKM), regulation of cell growth (IGFBP5, IGFBP2), positive regulation of cell proliferation and cell division (PDGFA, VEGFA). To gain a better understanding of the gene networks in PVA‐treated hPSCs, we performed GO analyses. The results showed that up‐ and downregulated DEGs in the PVA group were enriched in Top10 GO terms associated with molecular function, cell components and biological processes. The biological processes are mainly related to canonical glycolysis process, fructose metabolic process, oxidation‐reduction process and growth factor activity, respectively (Figure 7C). KEGG pathway analysis showed enrichments for glycolysis or gluconeogenesis, biosynthesis of amino acids, PI3K‐Akt signalling pathway, carbon metabolism and metabolic pathways (Figure 7D). Cellular metabolism is fundamental to all biological activities and is now known to play a pivotal role in dictating whether a cell proliferates, differentiates or remains quiescent.^42^ Recent studies of metabolism in stem cells have revealed energy metabolism such as glycolysis, oxidation–reduction process and oxidative phosphorylation must occur in order for cells to acquire sufficient nutrients such as glucose, amino acids, lipids and nucleotides that are necessary to support cell proliferation.^43^, ^44^ Taken together, these results demonstrated that the use of PVA could significantly promote hPSC proliferation through improving energy metabolism‐related processes, regulating cell growth, cell proliferation and cell division.

**FIGURE 8:**
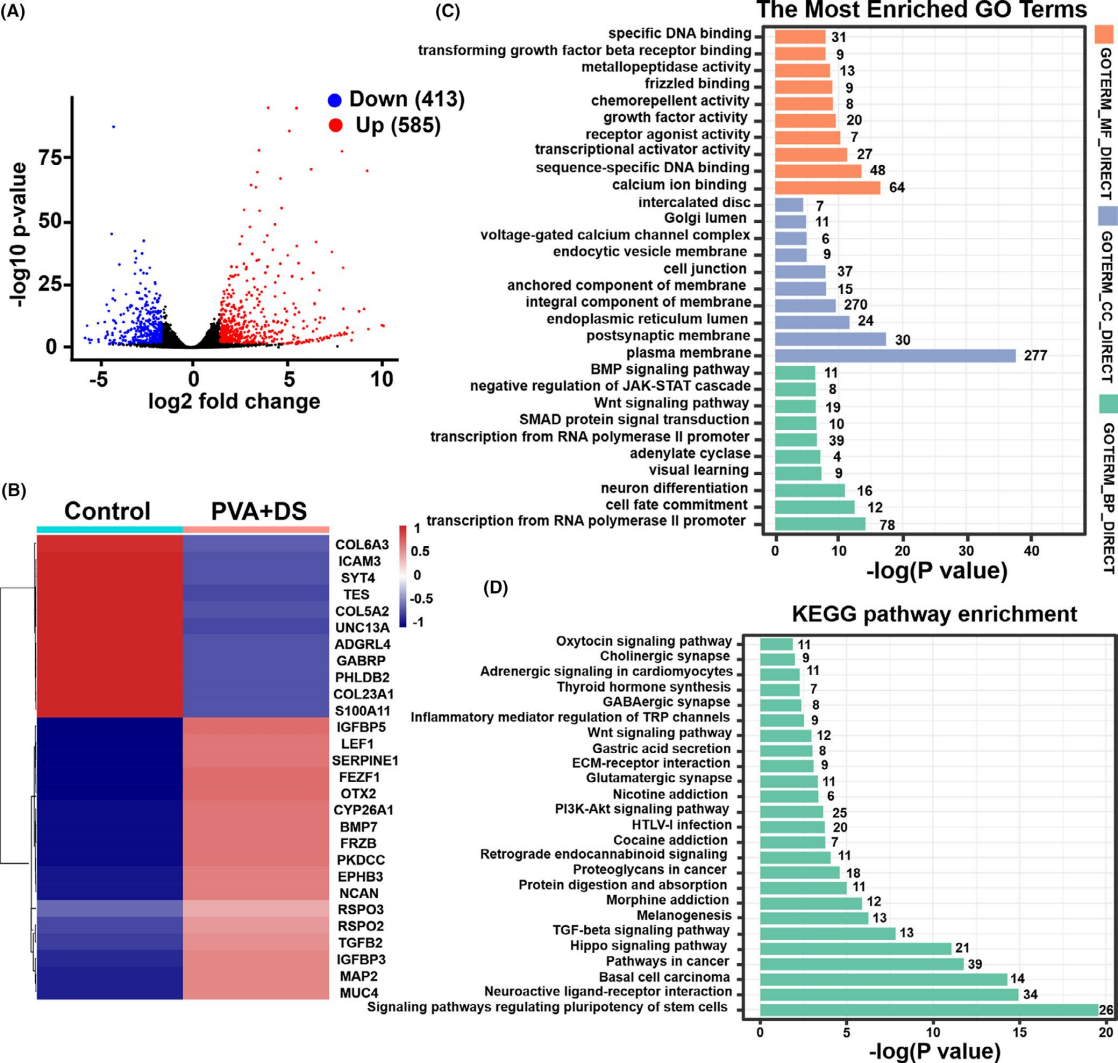
Transcriptome profiles of PVA and DS on hPSCs in static suspension culture. (A), Volcano map of DEGs of hPSCs in PVA + DS vs Control. Red dots indicate significantly upregulated DEGs; blue dots indicate significantly downregulated DEGs; and grey dots indicate no significance. (B), Heatmap of DEGs of hPSCs among Control and PVA + DS. (C), GO terms of PVA + DS groups. (D), KEGG pathway enrichment of hPSCs among Control and PVA + DS. Black number is the number of enriched genes

Finally, we compared the gene expression profiling between the Control and PVA plus DS groups. 413 downregulated and 585 upregulated DEGs were induced in hPSCs treated with a combination of PVA and DS. (Figure 8A). The heatmap of DEGs between the control and PVA plus DS group is shown in Figure 8B. It shows that PVA plus DS treatment had a significant effect on the transcriptomic profile. GO term enrichment analysis was further performed. Not surprisingly, most of the enriched GO terms are related to cell fate commitment, plasma membrane, integral component of membrane, cell junction, growth factor activity, Wnt and BMP signalling pathway (Figure 8C). KEGG pathway analysis was also carried out, and the result is shown in Figure 8D. The top pathways include signalling pathways regulating pluripotency of stem cells, Hippo signalling pathway, TGF‐beta signalling pathway, PI3K‐Akt signalling pathway and Wnt signalling pathway. These results indicated that a combined use of PVA and DS significantly promoted hPSC proliferation through improving energy metabolism‐related processes, regulating cell growth, cell proliferation, cell division and reducing the adhesion among hPSC aggregates by affecting expression of genes related to cell adhesion.

## 4 CONCLUSION

In the present work, we developed a chemical‐based method to control cell aggregation and also significantly enhance cell proliferation in hPSC suspension culture. Our results identified that supplementation of DS enables formation of reproducible, homogeneous and controlled hPSC aggregates without losing their pluripotency. Additionally, PVA treatment significantly promoted hPSC proliferation through improving energy metabolism‐related processes. A combination of DS and PVA offers two benefits by forming small aggregates and enhancing cell proliferation. This method might be an improvement over other existing methods because the recipes described here are simple and at low cost, facilitating use in large‐scale suspension cultures. In this present study, the effect of molecular weight of PVA on hPSC proliferation was not directly described, and further study is required to clarify. In addition, improvements might be made by using a more simply basal medium (eg. E8 or E6), instead of mTeSR1 which contains bovine serum albumin making it not a good candidate to see the effects of PVA and DS. In future research, it will be interesting to investigate whether we can integrate cell expansion and differentiation towards specific lineages (eg cardiomyocytes or hepatocyte) in a single spinner.

## CONFLICT OF INTEREST

The authors declare no conflict of interest.

## AUTHOR CONTRIBUTIONS

XLT conceived and designed the experiments, collected and analysed data, and prepared the manuscript. HBW was responsible for the experiment, figures and data analysis. JHX was responsible for the collection and analysis of the data. NW, QCC, ZYZ, YQQ, JW, XJL and PST contributed to the collection of the data. LXL provided materials. MA Z reviewed and revised the manuscript. YYD and HLC conceived and designed the experiments, and provided financial support.

## Supporting information

Supplementary Material

## Data Availability

The data that support the findings of this study are available from the corresponding author upon reasonable request.
