# Supplementary material for: The combination of dextran sulphate and polyvinyl alcohol prevents excess aggregation and promotes proliferation of pluripotent stem cells in suspension culture
Source: Cell Prolif. 2021 Aug 13;54(9):e13112. doi: 10.1111/cpr.13112 (PMC8450127; doi:10.1111/cpr.13112)
Supplement: Supplementary file 1 — Supplementary Material [file CPR-54-e13112-s001.docx]

**Supplemental Information**


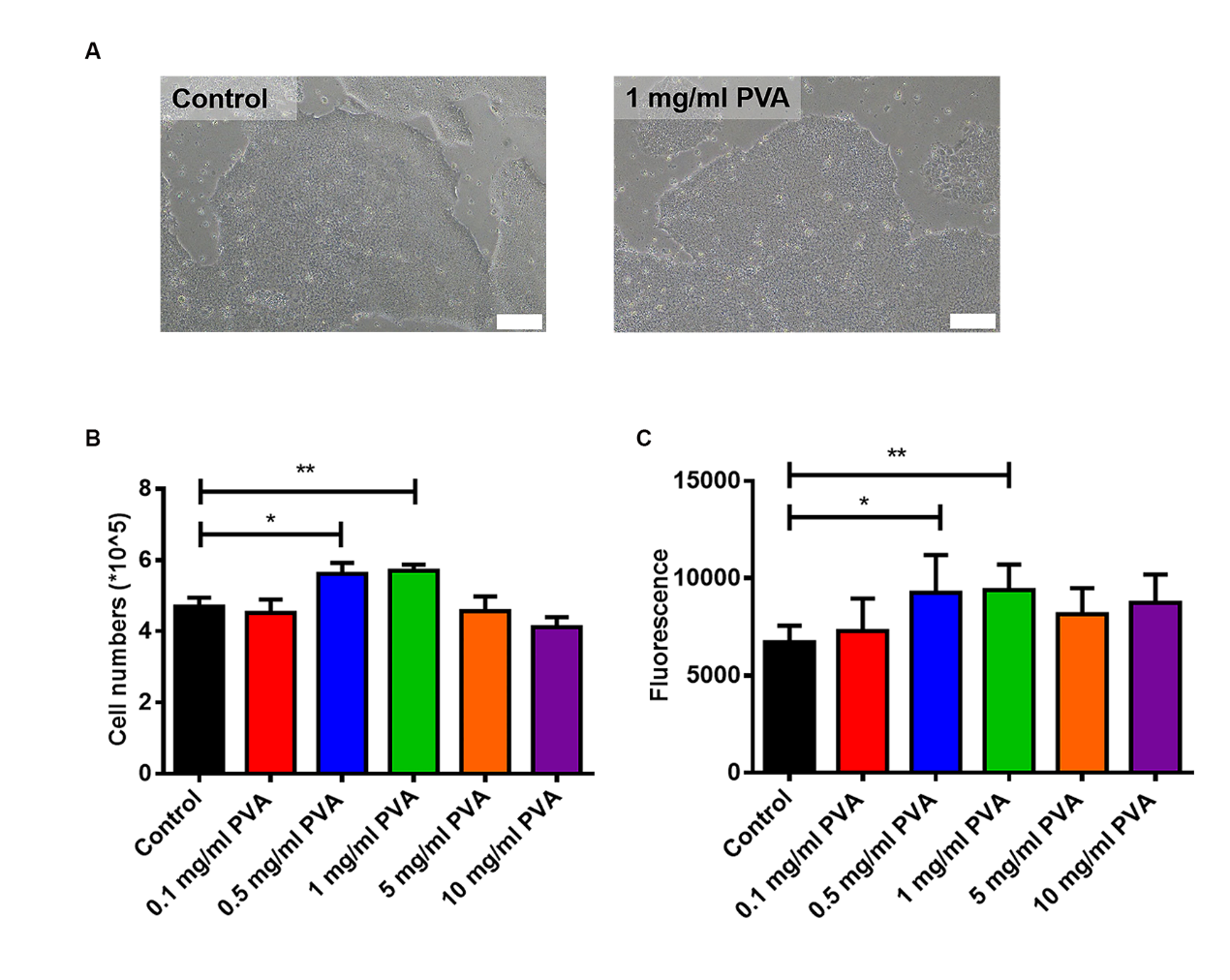


Figure S1. The effect of PVA on hiPSCs in 2D adherent culture. (A) Representative images of hiPSCs on day 5 in 2D adherent culture supplemented with 1 mg/ml PVA, scale bar = 200 μm. (B) Total numbers of hiPSCs on day 5 in 2D adherent culture supplemented with various PVA concentrations. (C) Cell viability of hiPSCs on day 5 in 2D adherent culture supplemented with various PVA concentrations. *P < 0.05, **P <0.01.


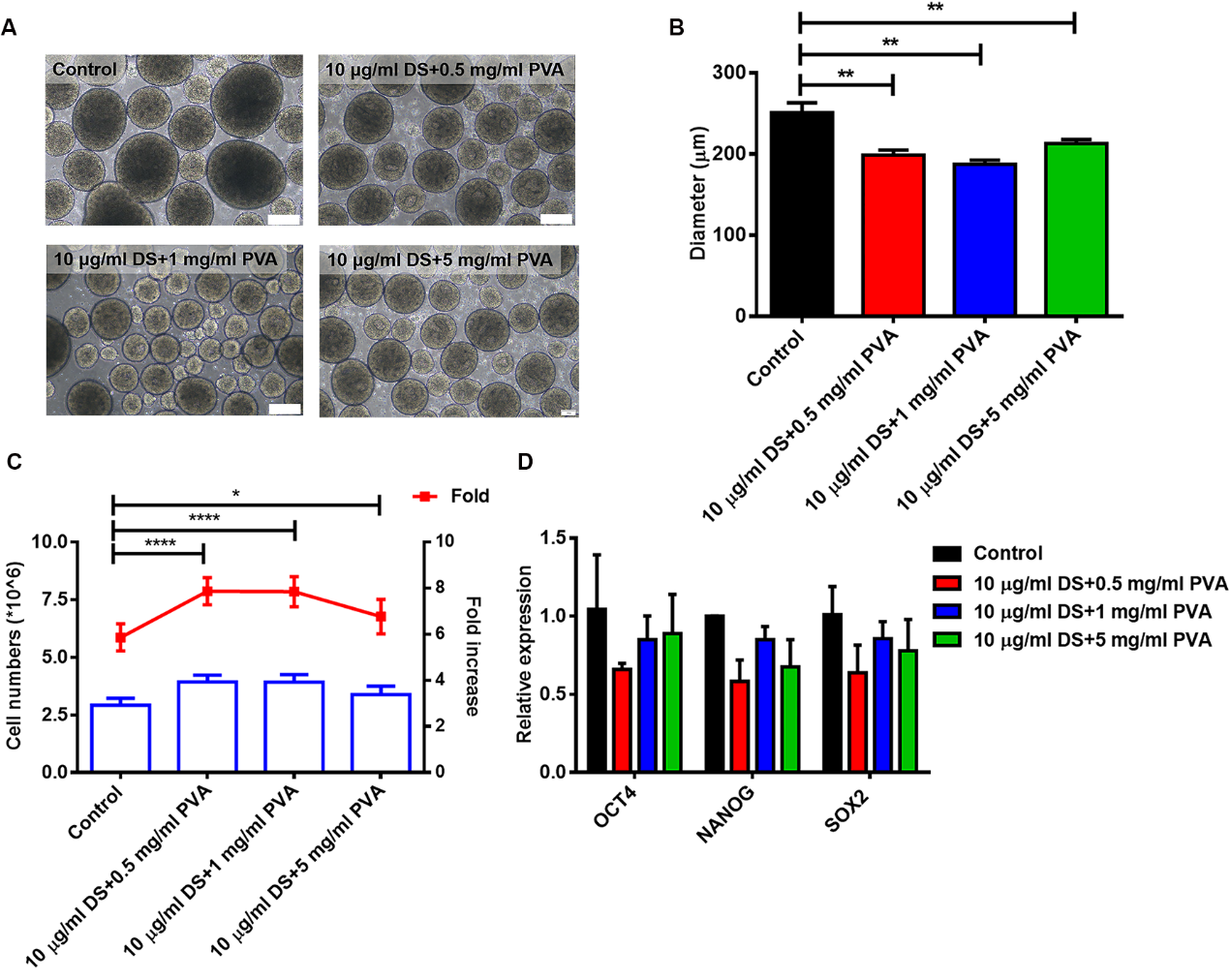


Figure S2. Results of hiPSCs in static suspension culture supplemented with DS (10 μg/ml) and PVA (0.5-5 mg/ml). (A) Representative images of hiPSC aggregates after 5 days of culture. Scale bar = 200 μm. (B) Average diameters of aggregates. (C) Comparison of cell yields after 5 days of culture. (D) Gene expression analysis by qPCR for pluripotent genes, OCT4, NANOG, and SOX2 after 5 days of culture. *P<0.05, **P<0.01, ***P<0.001.


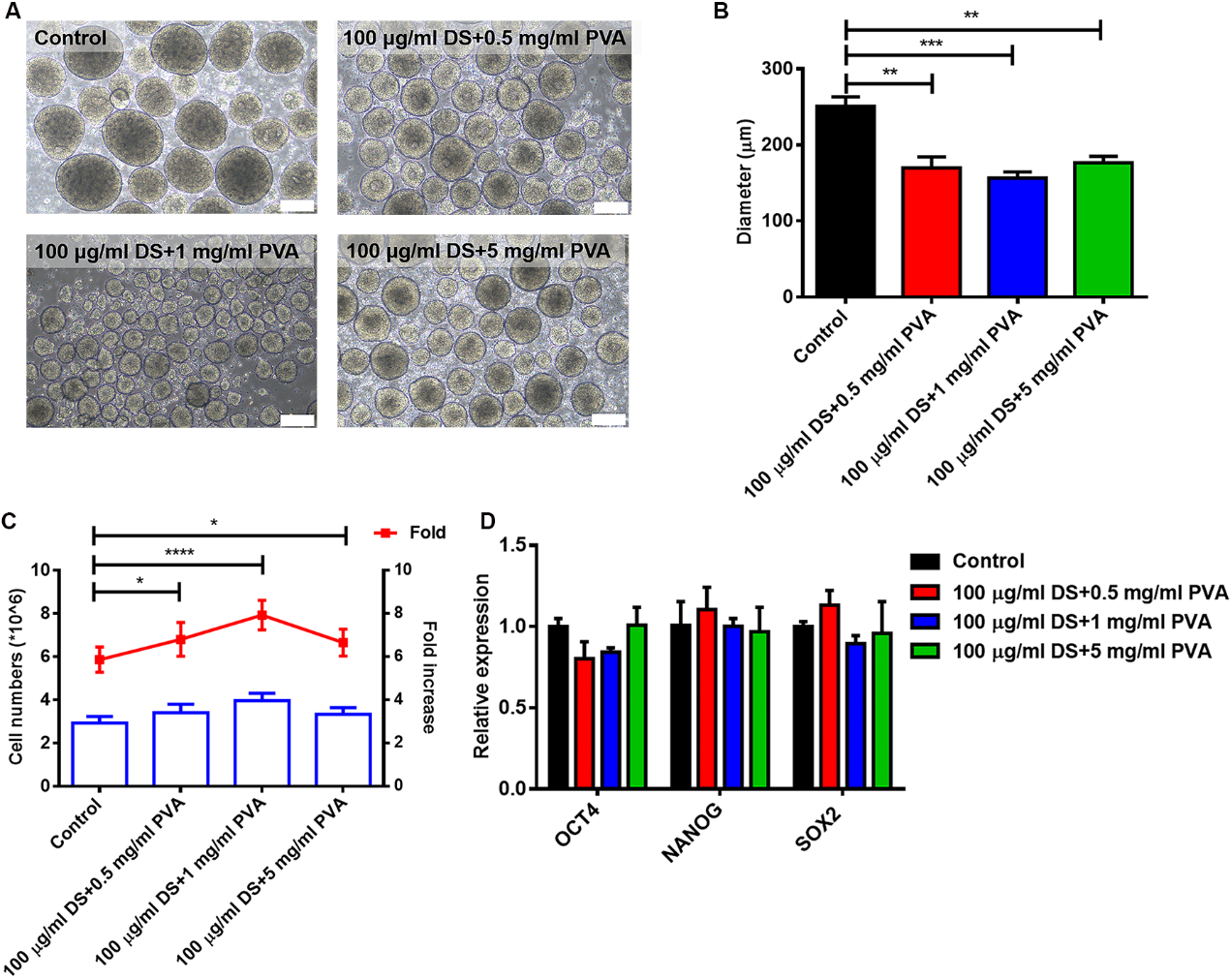


Figure S3. Results of hiPSCs in static suspension culture supplemented with DS (100 μg/ml) and PVA (0.5-5 mg/ml). (A) Representative images of hiPSC aggregates after 5 days of culture. Scale bar = 200 μm. (B) Average diameters of aggregates after 5 days of culture. (C) Comparison of cell yields after 5 days of culture. (D) Gene expression analysis by qPCR for pluripotent genes, OCT4, NANOG, and SOX2 after 5 days of culture. *P<0.05, **P<0.01, ***P<0.001.


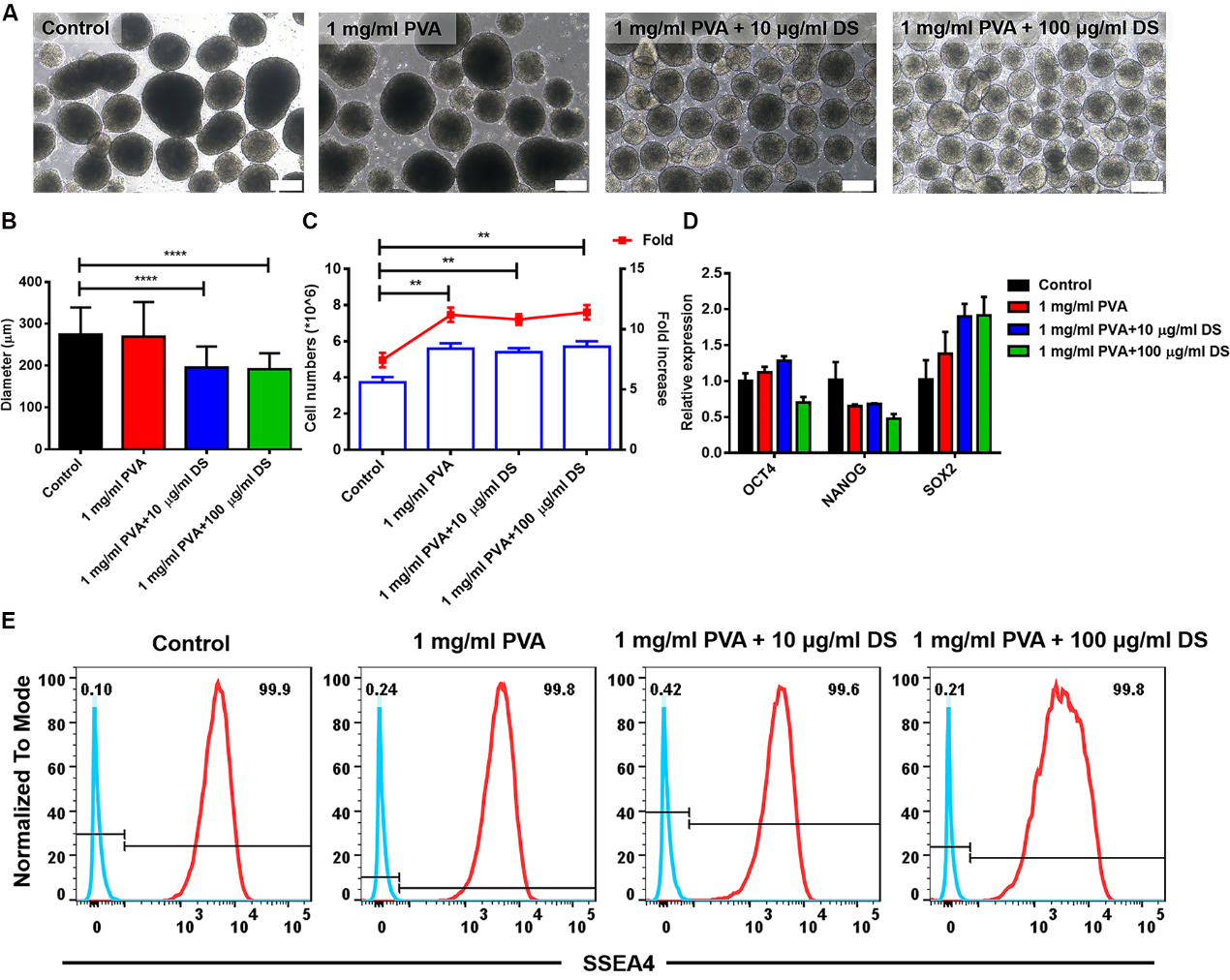


Figure S4. Results of H9 in static suspension culture supplemented with 1 mg/ml PVA and 100 μg/ml DS. (A) Representative images of H9 aggregates after 5 days of culture. Scale bar = 200 μm. (B) Comparison of average diameters of aggregates after 5 days of culture. (C) Comparison of cell yields after 5 days of culture. (D) Gene expression analysis by qPCR for pluripotent genes, OCT4, NANOG, and SOX2 after 5 days of culture. (E) Quantification of H9 aggregates expressing pluripotent marker SSEA4 using Flow cytometry analysis. **P<0.01, ***P<0.001.


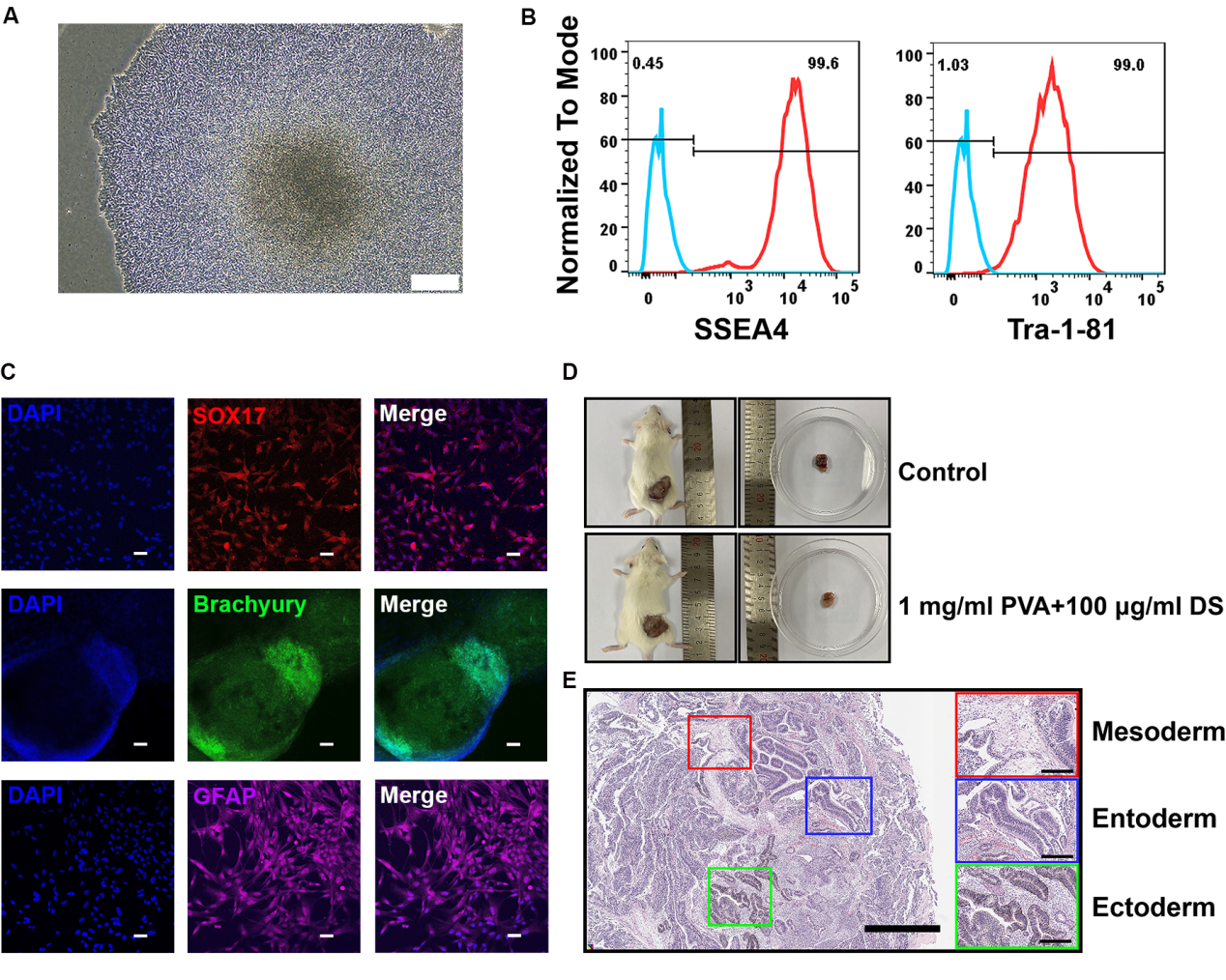


Figure S5. Expansion of hiPSCs in 3D dynamic suspension culture supplemented with 100 μg/ml DS and 1 mg/ml PVA. (A) Cell morphology of hiPSCs after being transferred back to 2D adherent culture system. Scale bar = 200 μm. (B) Flow cytometry analyses of the pluripotency of hiPSCs after being transferred back to 2D adherent culture system. (C) Immunofluorescence staining of embryoid bodies on Matrigel-coated plates. hiPSCs in 3D dynamic suspension culture treated with 100 μg/ml DS and 1 mg/ml PVA were left to form EBs for 2 weeks, and then were transferred onto Matrigel-coated plates. Cells were stained for three germ layer markers GFAP (ectoderm), Brachyury (mesoderm) and SOX17 (endoderm), scale bars = 50 μm. (D) Assessment of differentiation capacity of hPSCs in dynamic 3D suspension culture supplemented with 100 μg/mL DS and 1 mg/mL PVA. Teratoma formation in immunodeficient mice. (E) H&E staining of teratoma section showing three germ lineage tissues in the teratoma, scale bar = 1 mm (left) or 200 μm (right).


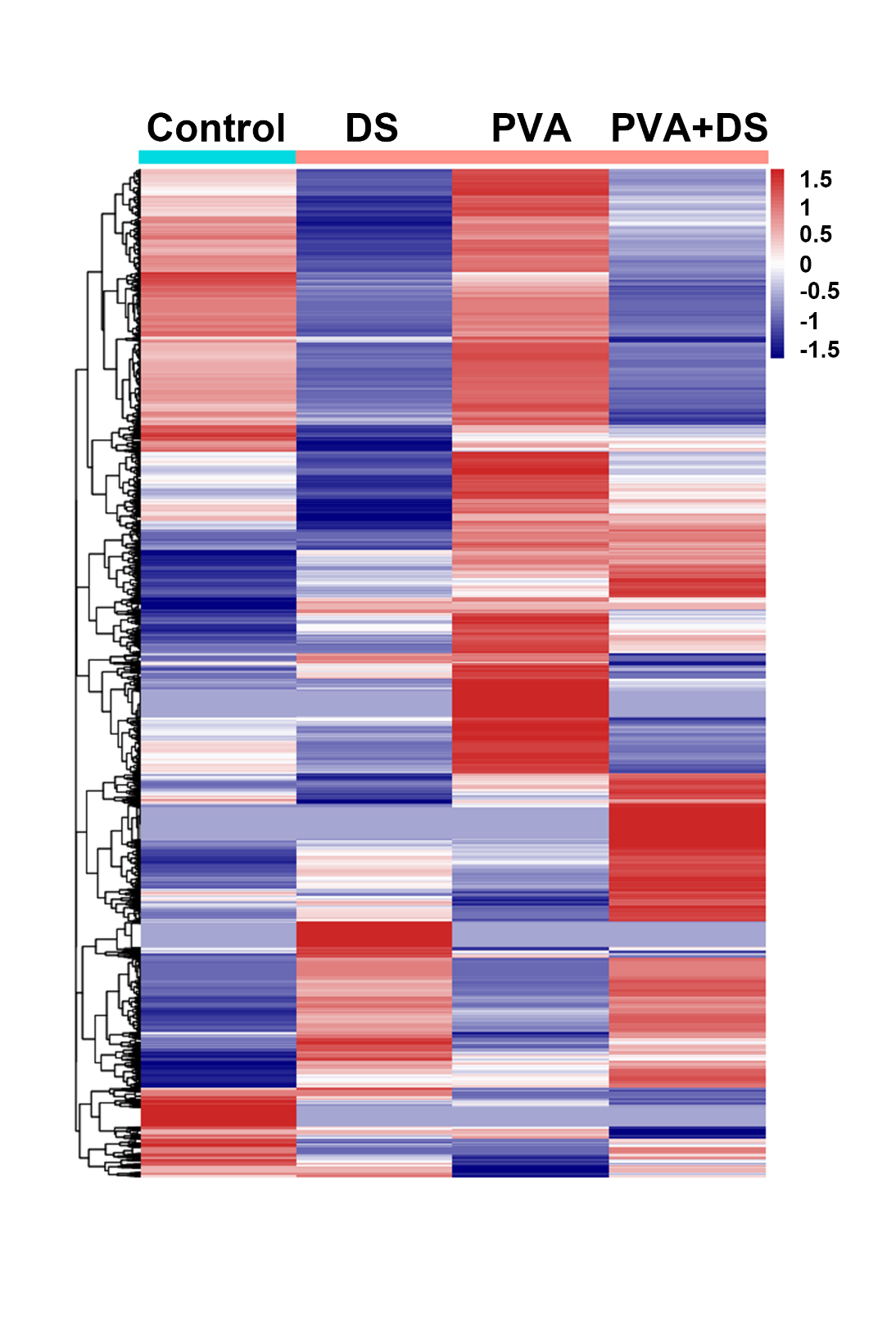


Figure S6. Transcriptome profiles of on hPSCs in static suspension culture. Heatmap of total genes of hPSCs among Control, DS, PVA and PVA plus DS.
